# Dehydration of Glucose to 5‐Hydroxymethylfurfural Using Nb‐doped Tungstite

**DOI:** 10.1002/cssc.201600649

**Published:** 2016-08-05

**Authors:** Chaochao Yue, Guanna Li, Evgeny A. Pidko, Jan J. Wiesfeld, Marcello Rigutto, Emiel J. M. Hensen

**Affiliations:** ^1^ Inorganic Materials ChemistryEindhoven University of TechnologyP.O. Box 5135600 MBEindhovenThe Netherlands; ^2^ Shell Global Solutions International B.V.P.O. Box 380001030 BNAmsterdamThe Netherlands; ^3^ King Abdullah University of Science and TechnologyKAUST Catalysis Center, Physical Sciences and Engineering Division, 4700 KAUSTThuwal23955-6900Saudi Arabia

**Keywords:** 5-hydroxylmethylfurfural, dehydration, glucose, niobium oxide, tungsten oxide

## Abstract

Dehydration of glucose to 5‐hydroxymethylfurfural (HMF) remains a significant problem in the context of the valorization of lignocellulosic biomass. Hydrolysis of WCl_6_ and NbCl_5_ leads to precipitation of Nb‐containing tungstite (WO_3_⋅H_2_O) at low Nb content and mixtures of tungstite and niobic acid at higher Nb content. Tungstite is a promising catalyst for the dehydration of glucose to HMF. Compared with Nb_2_O_5_, fewer by‐products are formed because of the low Brønsted acidity of the (mixed) oxides. In water, an optimum yield of HMF was obtained for Nb–W oxides with low Nb content owing to balanced Lewis and Brønsted acidity. In THF/water, the strong Lewis acidity and weak Brønsted acidity caused the reaction to proceed through isomerization to fructose and dehydration of fructose to a partially dehydrated intermediate, which was identified by LC‐ESI‐MS. The addition of HCl to the reaction mixture resulted in rapid dehydration of this intermediate to HMF. The HMF yield obtained in this way was approximately 56 % for all tungstite catalysts. Density functional theory calculations show that the Lewis acid centers on the tungstite surface can isomerize glucose into fructose. Substitution of W by Nb lowers the overall activation barrier for glucose isomerization by stabilizing the deprotonated glucose adsorbate.

## Introduction

Driven by growing environmental concerns about the negative impact of the combustion of finite fossil resources, significant efforts are currently underway to develop routes to fuels and chemicals based on renewable feedstock. Lignocellulosic biomass is considered as one of the most promising sources of renewable carbon for the sustainable production of fuels and chemicals.^1^ It is envisioned that the conversion processes in biorefineries will involve a limited number of key intermediates (platform molecules) with a wide range of downstream applications.^2^ The production of such platform molecules from cellulose, hemicellulose, and lignin needs to be performed with good efficiency. In this context, 5‐hydroxymethylfurfural (HMF), which can be obtained from glucose (the main constituent of cellulose) is considered to be one of the most versatile platform chemicals. Accordingly, the development of efficient catalysts for glucose dehydration has been well studied. A complete review on this topic is available.^3^ In general, to achieve a high yield of HMF, isomerization of glucose to its more reactive fructose isomer is required prior to dehydration of the sugar. Although the xylose isomerase enzyme is commercially used for the production of high‐fructose syrups, the main reason to develop heterogeneous catalysts is the limited compatibility of enzymes with the acidic conditions necessary for the subsequent sugar dehydration step. Both base and Lewis acid catalysts have been explored for this purpose.^3^ Lewis acids are preferred because their activity is not affected by strong Brønsted acids.^4^ Selective dehydration of fructose to HMF can be readily achieved in the presence of Brønsted acid catalysts in biphasic organic–water solvents.^5^, ^6^, ^7^

In addition to this two‐step approach, one‐pot strategies to directly convert glucose to HMF have gained attention recently. As indicated above, it is generally assumed that this reaction proceeds via fructose as an intermediate, although a recent investigation by Noma et al. indicated that a direct glucose dehydration pathway should also be considered.^8^ Lewis acid metal chlorides in ionic liquids or metal oxides in water can convert glucose to HMF in good yields.^9^, ^10^, ^11^ The use of the CrCl_2_/1‐ethyl‐3‐methylimidazolium chloride system under relatively mild conditions showed the promise of Lewis acids in ionic liquid solvents.^9^ Water‐tolerant Sn‐Beta zeolite and a Brønsted acid catalyst, such as HCl, form an effective combination to obtain HMF in good yield from glucose, especially in biphasic systems.^5^ A mechanism based on isomerization and dehydration was also proposed by Ståhlberg and co‐workers for the boric acid catalyzed glucose dehydration in ionic liquids.^12^ Furthermore, early transition metal oxides such as TiO_2_ and Nb_2_O_5_ are abundant and cheap materials with water‐tolerant and tunable acid–base properties with promising performance in sugar conversion reactions.^8^, ^13^, ^14^, ^15^ Among these, niobic acid (hydrated niobium pentoxide, Nb_2_O_5_⋅H_2_O) has received considerable attention.^13^, ^14^, ^15^ To obtain a good HMF yield, the strong Brønsted acidity of niobic acid needs to be reduced, for example, by phosphation.^13^ Similarly, it has been shown that modification of the acid–base properties of zirconia and tantalum oxide by sulfation or phosphation can improve the yields of HMF.^16^, ^17^, ^18^

The details of the mechanism of glucose conversion to HMF have already been discussed for a long time. Recently, important contributions from computational chemistry have shed new light on this topic.^19^, ^20^, ^21^, ^22^ The aldose–ketose isomerization is thought to occur either by proton transfer or by intramolecular hydride transfer. In the presence of a base, the aldose is deprotonated and the isomerization proceeds through a series of enolate intermediates followed by re‐protonation. In addition to fructose, this also yields mannose as the product of epimerization. Lewis acids catalyze the intramolecular hydride shift that transforms the open form of glucose to the open form of fructose.^20^ Dehydration of fructose is facile and removes three water molecules. Some of the intermediates have been identified.^23^, ^24^

In this work, we focus on tungstite as a potential catalyst for the dehydration of glucose to HMF. The structure of tungstite (WO_3_⋅H_2_O) is characterized by distorted octahedral units of tungsten atoms coordinating to five oxygen atoms and a water molecule. The octahedra share four corners in the equatorial plane to form sheets. Its surface mainly contains strong Lewis acid W^6+^ sites. We introduced Nb in the synthesis to modify the acidic properties and achieve a high HMF yield. A salient finding in the present study is that tungstite exhibits very weak Brønsted acidity, slowing down the complete dehydration of sugars to HMF. As a consequence, a partially dehydrated reaction intermediate was isolated by LC‐ESI‐MS. The addition of HCl rapidly converts this intermediate into HMF. This paper is organized as follows. In the first part, we present the characterization of the different oxides and their performance in glucose conversion in water. The optimum HMF yield is obtained at an intermediate Nb content as a result of balanced Lewis and Brønsted acidity. In the second part, density functional theory (DFT) calculations are used to demonstrate how Lewis acidic W and Nb sites catalyze glucose isomerization. After exploring various biphasic solvent mixtures, in the final part we show that the low Brønsted acidity in tetrahydrofuran (THF)/water mixtures limits the complete dehydration of glucose to HMF. In a two‐step approach involving HCl to dehydrate the intermediates, the HMF yield obtained from glucose by all of the tungstite catalysts is similar. This study provides insight into the mechanism of glucose dehydration and shows a novel approach to dehydrate glucose to HMF starting from weakly Brønsted acidic tungstite.

## Experimental Section

### Synthesis of materials

Mixed niobium–tungsten oxides, denoted by Nb_*x*_–WO_3_ where *x* indicates the Nb/W ratio (*x*=0.033, 0.1, 0.2, and 1), and tungsten oxide were prepared by stirring appropriate amounts of NbCl_5_ and WCl_6_ in water at room temperature. The light green precipitate that formed during stirring for 8 h was filtrated and washed with deionized water until the filtrate had a neutral pH. The samples were finally dried overnight at 60 °C. P/WO_3_ and P/Nb_0.2_–WO_3_ were prepared by stirring a suspension of the parent oxide in a solution of H_3_PO_4_ (1 g/200 mL) at room temperature for 2 days. The samples were retrieved by filtration and then washed with deionized water repeatedly until the pH of the filtrate was 6. These samples were dried at 60 °C for 10 h.

### Characterization

Powder X‐ray diffraction (XRD) patterns were measured on a Bruker D4 Endeavor powder diffraction system using CuK_α_ radiation. Nitrogen sorption was measured on a Micromeritics Tristar 3000 system in static measurement mode at−196 °C. The samples were outgassed at 120 °C for 3 h prior to the sorption measurements. The Brunauer–Emmett–Teller (BET) equation was used to calculate the specific area from the adsorption data (*p*/*p*_0_=0.05–0.25).

FTIR spectroscopy of adsorbed CO was used to evaluate the acid properties of Nb_*x*_–WO_3_ and P/Nb_*x*_–WO_3_. Infrared spectra (1200–4000 cm^−1^) were recorded in transmission mode in a Bruker Vertex V70v FTIR spectrometer equipped with a deuterated triglycine sulfate detector. The catalyst was pressed into a self‐supporting wafer with a diameter of 13 mm and then placed in a controlled atmosphere transmission cell equipped with CaF_2_ windows. Prior to CO adsorption, the sample was evacuated for 2 h at 50 °C with the pressure in the cell below 2×10^−6^ mbar. The sample was then cooled to −196 °C by flowing liquid N_2_ through a capillary spiraled around the catalyst wafer. An initial spectrum was recorded at this stage. CO was dosed through a sample loop connected to a six‐way valve. In this manner, accurate doses of 0.04 μmol CO were administered to the cell. Each IR spectrum was recorded by accumulating 64 scans at a resolution of 2 cm^−1^. Difference spectra were obtained by subtracting the initial spectrum of the treated catalyst from the spectra obtained at increasing CO coverage.

The CO stretch region of the various spectra was used to determine the density of the Lewis acid sites (LAS) and Brønsted acid sites (BAS). The extinction coefficient is assumed to be constant over the narrow frequency range of 2150–2200 cm^−1^. The value of the molar extinction coefficient was 2.7 cm/μmol.^25^ This value and the intensity of the relevant band were used to calculate the number of LAS and BAS using the Lambert–Beer law [Eq. (1)]:

(1) $$N=\frac{A}{\epsilon \rho },$$

in which *N* is the density of the vibrating species (mmol g^−1^), *A* is the intensity of the band (cm^−1^), ϵ is the molar extinction coefficient of CO (cm μmol^−1^) and ρ is the wafer thickness (mg cm^−2^).

### Catalytic activity measurements

Glucose conversion experiments were performed in a closed, magnetically stirred glass reactor under autogenous pressure at 120 °C. The reaction mixture consisted of 1 mL of an aqueous 1 wt % glucose solution in which 0.1 g catalyst was suspended. After the reaction, the mixture was quenched in water and the liquid part was analyzed by a Shimadzu HPLC system with evaporative light scattering detector (ELSD) and UV detectors. Glucose, fructose, and mannose were detected by ELSD using a Prevail Carbohydrate ES (Grace) column for separation. For sugars, the mobile phase (1.0 mL min^−1^) was acetonitrile/water (70:30 v/v) and the column temperature was 50 °C. HMF was detected by a UV detector (320 nm) with a Pathfinder PS C18 reversed phase column. The mobile phase (0.4 mL min^−1^) in this case was methanol/water (20:80 v/v) and the column temperature was 30 °C. The lactic acid concentration was determined by using a Prevail Organic Acid column in combination with a UV detector (220 nm). The mobile phase (0.5 mL min^−1^) was 25 mm potassium phosphate buffer with pH 2.5 and the column temperature was 45 °C.

Further analysis of the reaction mixtures was by LC‐MS (Agilent 1200). The LC‐MS was equipped with a diode array detector and autosampler, directly coupled to an ion trap mass spectrometer (Agilent ion trap 6320) through an electrospray interface. A Hypersil C18‐AR column was employed using acetonitrile (ACN) and water containing 0.1 % formic acid (water/FA) as the mobile phases, and was eluted according to the following gradient: 0 min, 98 % water/FA; 10 min, 80 % water/FA; 11 min, 100 % ACN; 13 min, 2 % water/FA. The flow rate was 0.2 mL min^−1^ and the injection volume was 5 μL. The diode array detector recorded spectra from 200 to 550 nm. The MS was operated under ESI negative ionization mode using the following parameters: dry temperature 350 °C, dry gas flow 9 L min^−1^, nebulizer gas pressure 40 psi, capillary voltage 3500 V. The instrument acquired data in the range of *m*/*z* 50–500.

### DFT calculations

All DFT calculations were performed by using the Vienna ab initio simulation package (VASP 5.3).^26^ The frozen‐core projector augmented wave (PAW) approach was employed to describe the interactions between core and valence electrons.^27^ The generalized gradient exchange‐correlation Perdew–Burke–Ernzerhof (PBE) functional and plane‐wave basis set with a cutoff energy of 500 eV were used.^28^ A Monkhorst–Pack mesh of 3×3×3 and 3×1×3 *k*‐points were used to sample the Brillouin zone for bulk and surface geometry optimization, respectively. The on‐site Coulomb correction for the W 5d states was included with a value of *U*_eff_=3.0 eV.^29^ The calculations were assumed to be converged when the forces on each atom were less than 0.05 eV Å^−1^, and the tolerance for energy convergence was set to 10^−5^ eV. Both the lattice vectors and atom positions were fully relaxed. The optimized lattice parameters for bulk orthorhombic WO_3_⋅H_2_O were found to be *a*=5.39 Å, *b*=11.03 Å, and *c*=5.19 Å. The WO_3_⋅H_2_O (010) surface model was employed to investigate the adsorption and isomerization of glucose by surface Lewis acid sites. A 2×1×2 supercell containing two layers of WO_3_⋅H_2_O and a vacuum slab of 15 Å along the (010) direction was employed. Each layer consisted of eight in‐plane WO_2_ units as well as four top and four bottom out‐of‐plane oxygen atoms and H_2_O molecules. Only the surface layer of WO_3_⋅H_2_O and the adsorbate were fully relaxed, whereas the other atoms and lattice parameters were fixed during the surface calculation. The closed cell electronic configuration of the WO_3_⋅H_2_O was determined as the electronic ground state.

The minimum reaction energy path and corresponding transition state were determined using the nudged elastic band method (NEB) with an improved tangent estimate.^30^ The maximum energy geometry along the reaction path obtained with the NEB method was further optimized using a quasi‐Newton algorithm. In this step, only the adsorbate was relaxed. Frequency analysis of the stationary points was performed by means of the finite difference method as implemented in VASP. Small displacements (0.02 Å) were used to estimate the numerical Hessian matrix. The transition states were confirmed by the presence of a single imaginary frequency corresponding to the reaction path. All the calculated energies were further corrected for dispersion interactions by single‐point energy calculations using the DFT‐D3 approach developed by Grimme and co‐workers.^31^

## Results and Discussion

### Characterization and glucose dehydration in water

**Figure 1:**
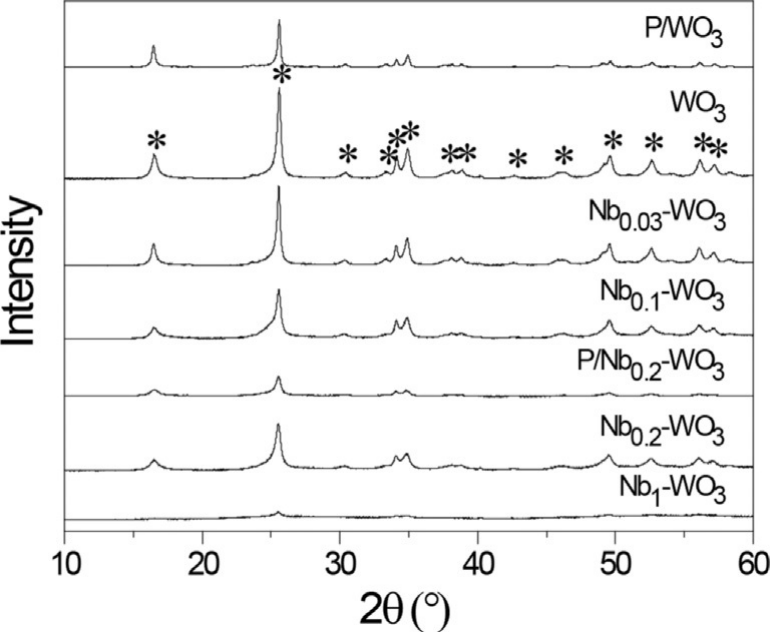
XRD patterns of Nb_*x*_–WO_3_ and WO_3_. The W‐containing oxides have the tungstite structure (✶ denotes tungstite, COD 9004174). XRD patterns of two phosphated samples are included.

Figure 1 shows the XRD patterns of the dried samples. All samples have the tungstite (WO_3_⋅H_2_O) structure^32^ independent of the Nb content. The crystallinity of the Nb_*x*_–WO_3_ oxides decreases with increasing Nb content. The crystallinity of the sample with Nb/W=1 is very low. The most important physicochemical properties of these samples are summarized in Table 1. The surface area of the mixed oxides increases with Nb content. The (commercial) amorphous niobic acid sample has the highest surface area. From this set, Nb_0.2_–WO_3_ with a high surface area and good crystallinity was selected to optimize the reaction conditions for the dehydration of glucose to HMF. Glucose isomerization was performed in single phase water at a temperature of 120 °C at a glucose concentration of 10 mg mL^−1^. After a 83 h reaction, the HMF yield was 34 % at a glucose conversion of 85 %. Higher initial glucose concentrations led to lower glucose conversion at nearly constant HMF selectivity (see Table 2). Table 3 shows that the glucose conversion and the HMF yield increase with reaction time. The highest HMF selectivity of 40 % was obtained after a 3 h reaction.

**Table 1:** Acid site densities of Nb_2_O_5,_ Nb_*x*_–WO_3_ and WO_3_ determined by IR spectroscopy of adsorbed CO.^[a]^

Catalyst	SA [m^2^ g^−1^]	*N*_BAS_ [μmol g^−1^]	*N*_LAS_ [μmol g^−1^]	LAS/BAS
Nb_2_O_5_	184	143	88	0.61
Nb_1_–WO_3_	160	82	35	0.43
Nb_0.2_–WO_3_	63	8.7	5.9	0.68
Nb_0.1_–WO_3_	42	11.8	8.8	0.74
Nb_0.03_–WO_3_	26	6.8	8.7	1.28
WO_3_	14	6.8	10.2	1.49

[a] SA=surface area, *N*_BAS_=number of BAS, *N*_LAS_=number of LAS.

**Table 2:** Dehydration of glucose to HMF using Nb_0.2_–WO_3_ in water (0.1 g catalyst, 1 mL H_2_O, 120 °C, 3 h).^[a]^

Glucose [mg mL^−1^]	*X*_sugar_ [%]	*Y*_HMF_ [%]	*S*_HMF_ [%]
100	34	12	35
50	37	13	35
10	86	34	40

[a] *X*_sugar=_sugar conversion, *Y*_HMF_=HMF yield, *S*_HMF_=HMF selectivity.

**Table 3:** Dehydration of glucose and fructose to HMF by using Nb_0.2_–WO_3_ (0.1 g catalyst, 10 mg glucose, 1 mL H_2_O, 120 °C).^[a]^

Substrate	*t* [h]	*X*_sugar_ [%]	*Y*_HMF_ [%]	*S*_HMF_ [%]
Glucose	1	68	16	24
	2.5	81	29	36
	3	86	34	40
	6	93	36	39
	12	93	31	33
Fructose	1	98	27	28
	2	100	30	30
	3	100	28	28

[a] *X*_sugar_=sugar conversion, *Y*_HMF_=HMF yield, *S*_HMF_=HMF selectivity.

**Figure 2:**
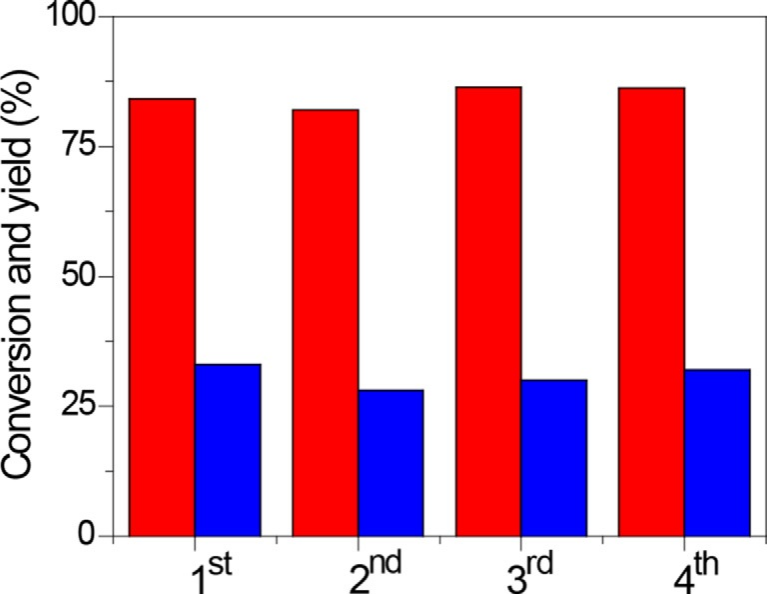
Reusability of Nb_0.2_–WO_3_ for the dehydration of glucose in water at 120 °C (red: glucose conversion; blue: HMF yield).

We then evaluated the performance of the Nb_*x*_–WO_3_ set and Nb_2_O_5_ at optimized conditions (120 °C, initial glucose concentration of 10 mg mL^−1^, a reaction time of 3 h). The results are shown in Table 4. The low HMF yield of 16 % at full glucose conversion for niobic acid is consistent with the work of Nakajima et al.^13^ The Nb_*x*_–WO_3_ samples afforded higher HMF yields under similar reaction conditions. Although Nb_1_–WO_3_ and Nb_0.2_–WO_3_ gave the highest and nearly similar HMF yields of approximately 35 %, the HMF selectivity was highest for Nb_0.2_–WO_3_. A further decrease of the Nb content resulted in lower glucose conversion and HMF selectivity. The reusability of the catalyst was also tested for Nb_0.2_–WO_3_. For this purpose, the spent catalysts were first washed with deionized water and then dried at 60 °C for 6 h. These recycle experiments demonstrate that high glucose conversion with only a small loss of the HMF yield was possible for at least four cycles (Figure 2).

**Table 4:** Dehydration of glucose to HMF using mixed oxides (0.1 g catalyst, 10 mg glucose, 1 mL H_2_O, 120 °C, 3 h).^[a]^

Catalyst	*X*_glucose_ [%]	*Y*_fructose_ [%]	*Y*_HMF_ [%]
Nb_2_O_5_	100	<0.1	16
Nb_1_–WO_3_	97	<0.1	35
Nb_0.2_–WO_3_	86	0.6	34
Nb_0.1_–WO_3_	83	0.5	30
Nb_0.03_–WO_3_	79	0.6	25
WO_3_	80	0.6	25

[a] X_glucose_: glucose conversion, Y_fructose_: fructose yield, Y_HMF_: HMF yield.

**Figure 3:**
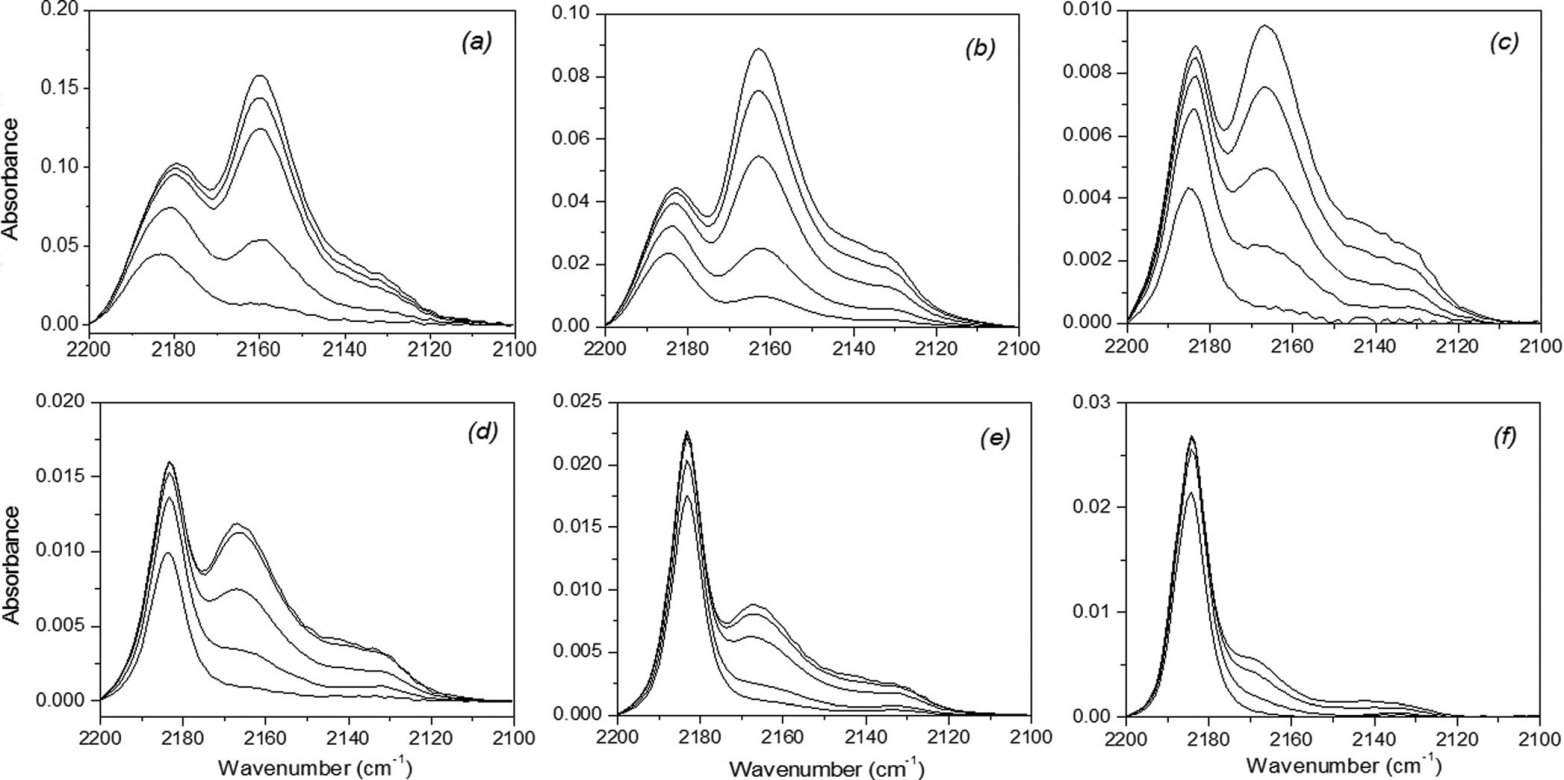
FTIR spectra of CO adsorption for (a) Nb_2_O_5_, (b) Nb_1_–WO_3_, (c) Nb_0.2_–WO_3_, (d) Nb_0.1_–WO_3_, (e) Nb_0.03_–WO_3_ and (f) WO_3_ (spectra recorded at 90 K; the various traces from bottom to top represent increasing CO coverage; the top trace is after complete saturation of the bands).

Although Brønsted acids are sufficient to convert fructose to HMF, selective conversion of glucose usually requires isomerization to fructose prior to dehydration, for which Lewis acids are effective.^5^, ^33^ Accordingly, we characterized the Brønsted and Lewis acid properties of the Nb_*x*_–WO_3_ and Nb_2_O_5_ samples by IR spectroscopy of adsorbed CO. Figure 3 shows the CO stretching region of the IR spectra. The spectra contain three bands at 2132 cm^−1^, 2160–2166 cm^−1^, and 2180–2185 cm^−1^, which are assigned to physisorbed CO, CO adsorbed on BAS, and LAS, respectively. It is immediately clear that the amount of BAS and LAS strongly increase with Nb content. For the Nb_*x*_–WO_3_ samples with *x*<1, the CO IR band attributed to BAS is located at 2166 cm^−1^, whereas this feature shifts to 2163 cm^−1^ for Nb_1_–WO_3_ and to 2160 cm^−1^ for Nb_2_O_5_. It is likely that the band in the Nb_1_–WO_3_ spectra is a composite owing to the presence of slightly acidic W−OH and Nb−OH groups. It should be noted that the acidity of niobic acid increases in the presence of water. Therefore, it might be that we do not probe all the acid sites that are present in the catalyst under working conditions. The BAS and LAS densities in these samples were estimated according to known procedures^25^ from the spectra after CO saturation and the results are listed in Table 1. The Nb_2_O_5_ sample contains much more BAS and LAS than the Nb_*x*_–WO_3_ and WO_3_ samples. The concentration of BAS and LAS of Nb_1_–WO_3_ are roughly half of the values of Nb_2_O_5_, whereas the other samples with much less Nb contain much less acid sites. The decrease in the total acidity with decreasing Nb content is much more pronounced than the decrease of the surface area. Thus, the changes in acidity can in part be attributed to the different chemical composition of the surface. Isomorphous substitution of W^6+^ by Nb^5+^ in tungstite will replace W^6+^−H_2_O by Nb^5+^−OH, which can explain the decrease of the concentration of LAS (cf. the decreasing ratio of LAS and BAS in Table 1). The Nb−OH groups are expected to be slightly basic. The weak BAS in these samples likely arise from hydroxyl groups present at the defect sites such as crystal edges. The samples may also contain niobic acid. The CO IR and textural data suggest that this is only the case for the Nb_1_–WO_3_ sample.

Taking into account these acidity differences, we can attribute the lower glucose conversion and higher HMF yield of the tungstite samples as compared with Nb_2_O_5_ to the decreased Brønsted acidity. The highest HMF selectivity was obtained with the Nb_0.2_–WO_3_ sample. Whereas the HMF selectivity is higher for the Nb_0.2_–WO_3_ sample, Nb_1_–WO_3_ can convert more glucose. The lower HMF selectivity for Nb_1_–WO_3_ is owing to strong acid sites originating from niobic acid. These strong BAS catalyze side reactions including oligomerization to humins, as evident from the reaction mixtures turning yellow to brown after a prolonged reaction, as well as retro‐aldol reactions that decompose sugars. In all of the reaction mixtures, a small amount of fructose was observed with a yield below 1 %. This demonstrates that fructose is a reaction intermediate and that the LAS in the Nb_*x*_–WO_3_ samples can isomerize glucose to fructose. The BAS catalyze the dehydration of fructose to HMF. We also attempted to use Nb_0.2_–WO_3_ as an isomerization catalyst by lowering the reaction temperature. However, at 100 °C the glucose conversion to HMF was less than 10 % and the fructose yield was below 1 %.

### DFT calculations on glucose isomerization

**Figure 4:**
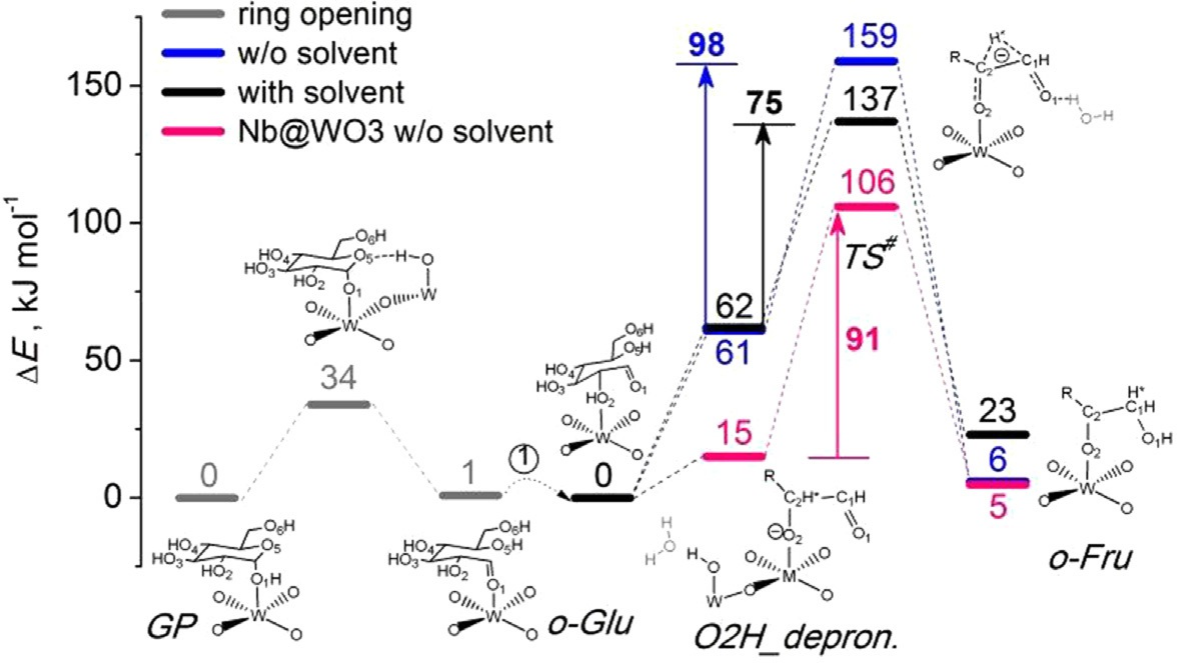
Reaction energy diagram for the isomerization of glucose on the (010) surface of WO_3_⋅H_2_O. For clarity, only the first coordination sphere of the central cation is shown. The central cation is W^6+^ or Nb^5+^ (all energies in kJ mol^−1^).

Because these preliminary data demonstrate that tungstite should be an active catalyst in glucose isomerization, we used DFT calculations to investigate possible reaction pathways for glucose isomerization on the stable (010) surface of orthorhombic WO_3_ as a model for the WO_3_⋅H_2_O surface. Based on earlier work,^19^, ^20^ we explored a mechanism involving three steps: (i) α‐d‐glucopyranose (*Glu*) ring opening to the acyclic form of glucose (*o‐Glu*), (ii) hydride shift from the C2 to the C1 atom in glucose, and (iii) ring closure of acyclic fructose (*o‐Fru*) to form α‐d‐fructofuranose (*Fru*). Glucose coordinates through its O1 atom sites to a surface tungsten site in a planar adsorption model (Figure 4). The ring opening of *Glu* is initiated by deprotonation of the O1H hydroxyl group, which involves the adjacent terminal O atom of W^6+^=O as a proton acceptor. This step generates an anionic *Glu* intermediate containing one negative charge located at the O1 atom and a reduced W−OH moiety in which the adjacent W atom has the 5+ oxidation state. The re‐protonation of the O5 atom of *Glu* leads to opening of the glucose ring. The proton in the resulting W−OH is then transferred to the O5 site. This step re‐oxidizes the W^5+^−OH surface intermediate back to the W^6+^=O state. The activation barrier for the ring‐opening step is only 34 kJ mol^−1^. After this step, the isomerization reaction takes place. To facilitate the O2H deprotonation process, *o‐Glu* has to change its coordination mode from O1−W coordination to O2H−W. Although this conformational change drastically disturbs the H‐bonding interaction of the hydroxyl groups of *o‐Glu* with the WO_3_ surface, we predict that such reorientation will proceed with a low barrier in aqueous solution because the solvent molecules provide efficient stabilization through the hydrogen‐bonded network. Deprotonation of O2H by W^6+^=O results in the anionic *o‐Glu* intermediate and a W^5+^−OH group. This step is endothermic by 61 kJ mol^−1^. The isomerization step itself proceeds through the intramolecular H‐shift from C2 to C1 with an activation barrier of 98 kJ mol^−1^. The terminal O1 atom of the anionic *o‐Fru* intermediate is then protonated by a co‐adsorbed H_2_O molecule coordinating to a nearby W^6+^=O (W^6+^=O⋅⋅⋅H_2_O). Finally, fructose is produced by ring closure. As solvent effects in these polar reactions are important,^19^ we also investigated the effect of additional water molecules on the O2H deprotonation and O1 protonation reactions. The activation barrier for the H‐shift reaction in the presence of more water decreased from 98 to 75 kJ mol^−1^. We then investigated the influence of substituting W with Nb on the WO_3_ surface. This substitution lowers the deprotonation energy of the O2H moiety to 15 kJ mol^−1^, whereas the barrier for the H‐shift reaction remains nearly unchanged (91 kJ mol^−1^). Thus, the overall barrier for the H‐shift reaction is lowered to 106 kJ mol^−1^ as compared to the value of 132 kJ mol^−1^ computed for the pure WO_3_ model surface. Qualitatively, the lower barrier for the mixed oxide surface is consistent with the higher glucose isomerization activity of the Nb_*x*_–WO_3_ mixed oxides in comparison to WO_3_. In summary, these computational modeling results show that glucose can be isomerized in water on the Lewis acid surface sites of WO_3_ and that Nb substitution on the WO_3_ surface facilitates sugar deprotonation, thereby lowering the overall activation barrier for isomerization.

### Effect of solvent on glucose dehydration

**Figure 5:**
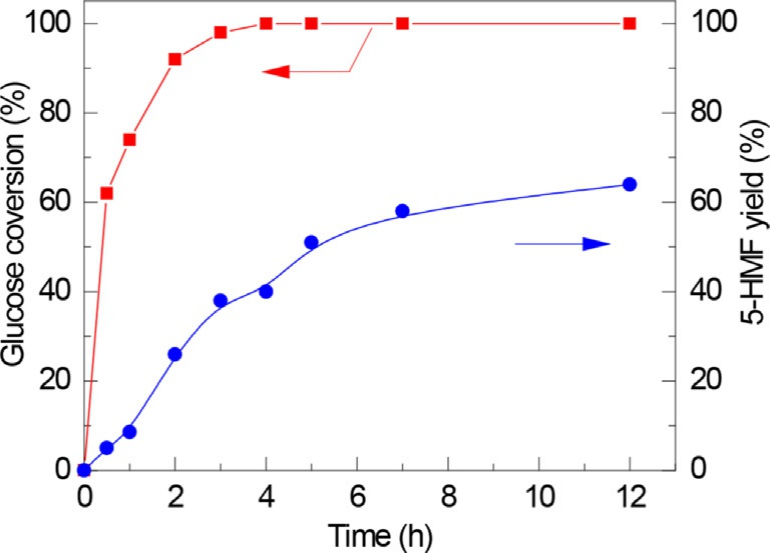
Transformation of glucose solution as a function of reaction time by using Nb_0.2_–WO_3_ in THF/H_2_O (90:10, v/v) at 120 °C (▪ glucose conversion; • HMF yield).

As it is well known that the use of other solvents and biphasic solvent systems can significantly increase the selectivity of glucose dehydration,^5^ we compared the performance of the optimum Nb_0.2_–WO_3_ sample in different solvents. The results are summarized in Table 5. In DMSO, the HMF yield is only 15 % at almost full conversion of glucose. The low HMF selectivity is possibly caused by glucose condensation reactions in anhydrous DMSO.^35^ In biphasic 1‐butanol/water,^5^ the HMF yield improved to 52 % after a 3 h reaction compared with the experiments in water. The increased performance is owing to the extraction of HMF to the organic layer, which protects it from further acid‐catalyzed side reactions in water such as the formation of humin. We also tested this catalyst in a mixture of THF/H_2_O in a 90:10 v/v ratio and found that with increasing reaction time the HMF yield increased only slowly. Figure 5 shows that, despite high initial glucose conversion, the HMF yield using Nb_0.2_–WO_3_ was only 6 % after 30 min. After a 3 h reaction, glucose conversion was nearly complete at a HMF yield of 38 %. However, after a 12 h reaction the HMF yield was 62 %. As the fructose yield was below 1 % during the whole experiment, we speculate that one of the dehydration steps towards HMF is very slow owing to the low Brønsted acidity of Nb_0.2_–WO_3_. To verify this, we used HCl to adjust the pH of the reaction mixture obtained after 3 h reaction to 1. After further reaction for 1 h at 120 °C, the HMF yield increased to 58 % (Table 6). At 5 and 10 times higher glucose starting concentration, the HMF yields were still 53 % and 29 %, respectively, if the experiment was performed in this two‐step approach. In the next section, we discuss the use of phosphate‐modified catalysts and LC‐ESI‐MS to identify the intermediate products.

**Table 5:** Dehydration of glucose to HMF in different solvents using Nb_0.2_–WO_3_ (0.1 g catalyst, 10 mg glucose, 1 mL solvent, 120 °C, 3 h).^[a]^

Solvent	*X*_glucose_ [%]	*Y*_HMF_ [%]
H_2_O	86	34
DMSO	100	15
1‐BuOH/H_2_O=3	98	52
THF/H_2_O=9	98	38
THF/H_2_O=9 (5 h)	100	51

[a] *X*_glucose_=glucose conversion, *Y*_HMF_=HMF yield.

**Table 6:** Dehydration of sugars to HMF using different catalysts in THF/water (0.1 g Nb_*x*_–WO_3_, 10 mg glucose, 1 mL H_2_O mixed with 9 mL THF, 120 °C).^[a]^

Substrate	Catalyst	*N*_BAS_ [μmol g^−1^]	*N*_LAS_ [μmol g^−1^]	LAS/BAS	*t* [h]	*X*_glucose_ [%]	*Y*_HMF_ [%]
Glucose	Nb_0.2_–WO_3_	8.7	5.9	0.68	3	100	38
	P/Nb_0.2_–WO_3_	15.2	13.4	0.88	3	100	30
	Nb_0.2_–WO_3_+HCl^[b]^	994	5.9	–	3	100	58
						100^[c]^	53^[c]^
						99^[d]^	29^[d]^
	WO_3_	6.8	10.2	1.49	3	100	10
	P/WO_3_	5.5	10.6	1.94	1	100	1
					2	100	2
					3	100	6
					4	100	6
					5	100	9
					5^[e]^	100	50
	P/WO_3_+HCl^[b]^	985	10.6	–	3	100	58
	HCl^[b]^	985	0	–	3	100	0
Fructose	Nb_0.2_–WO_3_	8.7	5.9	0.68	2	100	27
	P/Nb_0.2_–WO_3_	15.2	13.4	0.88	2	100	20
	WO_3_	6.8	10.2	1.49	2	100	12
	P/WO_3_	5.5	10.6	1.94	2	100	8
	HCl	985	0	–	2	100	64
	H_3_PO_4_	985	0	–	2	20	10

[a] *X*_glucose_=glucose conversion, *Y*_HMF_=HMF yield, *N*_BAS_=number of BAS, *N*_LAS_=number of LAS. [b] pH 1.0. [c] 50 mg Glucose. [d] 100 mg Glucose added. [e] After 2 h reaction, the catalyst was removed and HCl was added and the reaction was continued for 3 h.

### Mechanistic considerations

**Figure 6:**
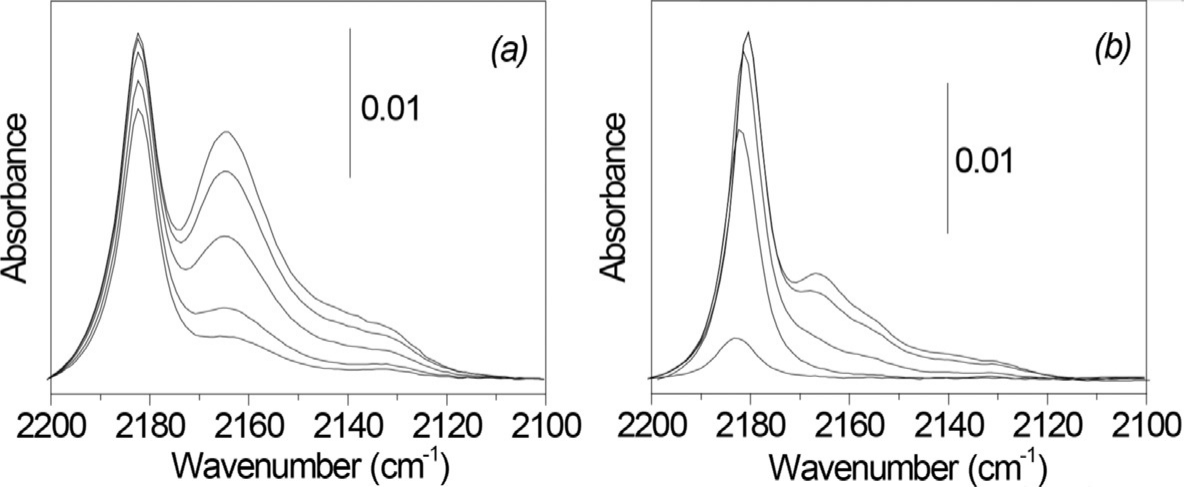
FTIR spectra of CO adsorbed to (left) P/Nb_0.2_–WO_3_ and (right) P/WO_3_ (spectra recorded at 90 K; the various traces represent increasing CO coverage; the top trace is after complete saturation).

In an attempt to gain an understanding of the role of the weak Brønsted acidity in the slow formation of HMF and the optimum conditions for the identification of possible reaction intermediates, we modified the acidity of Nb_0.2_–WO_3_ and WO_3_ using phosphoric acid. We included WO_3_ at this stage because we hypothesized that the lowest HMF yield obtained for the WO_3_ sample could be because of to the low acidity of this sample. Characterization of the phosphated samples by CO IR spectroscopy (Table 6 and Figure 6) confirmed that the ratio of LAS to BAS of these phosphated samples was higher than those of the parent materials. This explains the lower HMF yields of P/Nb_0.2_–WO_3_ and P/WO_3_ compared to the parent samples. For Nb_0.2_–WO_3_, the HMF yield decreased from 38 % to 30 %. The HMF yield for P/WO_3_ was much lower, despite the complete conversion of glucose after 3 h. It has been reported that deactivation of the BAS of Nb_2_O_5_, Ta_2_O_5_, and ZrO_2_ by phosphate groups increases the HMF yield during glucose conversion.^13^, ^16^, ^17^, ^18^ In our case, the decrease of the Brønsted acidity upon phosphation of Nb_0.2_–WO_3_ and WO_3_ results in decreased HMF yield, presumably because, different from the other materials, the rate‐controlling step is dehydration of a reaction intermediate. Consistent with this, we found that the addition of HCl to bring the pH of the solution to 1 followed by further reaction for 1 h at the same temperature resulted in a steep increase of the HMF yield to 58 %. These results underpin our assumption that the reaction mixtures contain a relatively stable reaction intermediate that can be dehydrated to HMF by HCl.

As these data imply that the Brønsted acidity of the tungstite samples is too weak to completely dehydrate glucose to HMF, and fructose is assumed to be an intermediate in the overall reaction, we also studied the dehydration of fructose by WO_3_ and Nb_0.2_–WO_3_ before and after phosphation (Table 6). Fructose conversion was complete after 2 h for all samples. This observation, together with the observation that phosphation decreased the HMF yield, suggests that similar reaction intermediates are formed during glucose and fructose dehydration. This result is also consistent with the low fructose yields observed during glucose dehydration, that is, isomerization of glucose to fructose and dehydration of fructose to the unknown intermediate are much faster than the further dehydration of the intermediate to HMF. The fructose conversion results also show that the BAS of (phosphated) tungstite are already sufficiently strong to initiate the dehydration of fructose. We also verified that the HMF yield during fructose conversion is very low if H_3_PO_4_ is used as a catalyst, which shows that the phosphate groups do not contribute to the conversion of fructose. On the contrary, a reasonable HMF yield was obtained from fructose with HCl.

**Figure 7:**
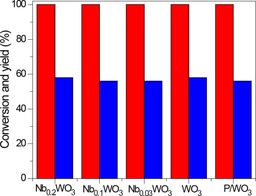
Catalytic performance for Nb_*x*_–WO_3_ after 3 h conversion of a solution of glucose in THF/H_2_O (90:10, v/v) by the combination of the catalysts and HCl (the solvent pH is 1). The reaction temperature was 120 °C (red: glucose conversion, blue: gray: HMF yield).

As the influence of phosphation was largest for WO_3_, we used the P/WO_3_ sample to study glucose dehydration in more detail. As the reaction proceeded, the HMF selectivity increased much slower than the glucose conversion (Table 6). Fructose yields were below 1 % in this experiment. After 5 h, the HMF yield was less than 10 %. In another experiment, we removed the P/WO_3_ sample from the reaction mixture after 1 h by filtration and adjusted the pH of the filtrate to 1 by HCl. This led to a fast increase of the HMF yield to 50 % after a 3 h reaction. We verified that mixing the glucose solution with an equivalent amount of HCl did not yield HMF, which is the expected result. These data further support the conclusion that dehydration of some reaction intermediates is slowed down by the weak acidity of the catalyst. We then reacted glucose in a THF/water mixture in the presence of the Nb_*x*_–WO_3_ with *x*<1 (the samples which did not contain niobic acid) for 3 h followed by adjustment of the pH to 1 and continued the reaction for 1 h at the same temperature. In all cases, the HMF yield was very similar at 56 %±2 % (Figure 7). Thus, we infer that the earlier differences in the HMF yields observed for the Nb_*x*_–WO_3_ samples are, in large part, related to acidity differences that lead to varying amounts of the unknown reaction intermediates dehydrating to HMF. In contrast to other catalyst systems, in which isomerization limits the reaction rate, the dehydration of the relatively stable reaction intermediates is most likely the rate‐limiting step on tungstite. This opens the possibility for a two‐step glucose conversion strategy consisting of isomerization on LAS and partial dehydration under weakly acidic conditions on BAS followed by dehydration of the intermediates by a mineral acid. This approach results in HMF yields of close to 60 %, which is comparable to other transition metal oxide systems,^8^, ^13^, ^14^, ^15^, ^16^, ^17^, ^18^ yet lower than results obtained with Sn‐Beta/HCl in THF/water.^5^ The advantage of the use of tungstite catalysts is that they can operate at lower temperature and the catalyst is much easier to prepare than Sn‐Beta.

**Scheme 1:**
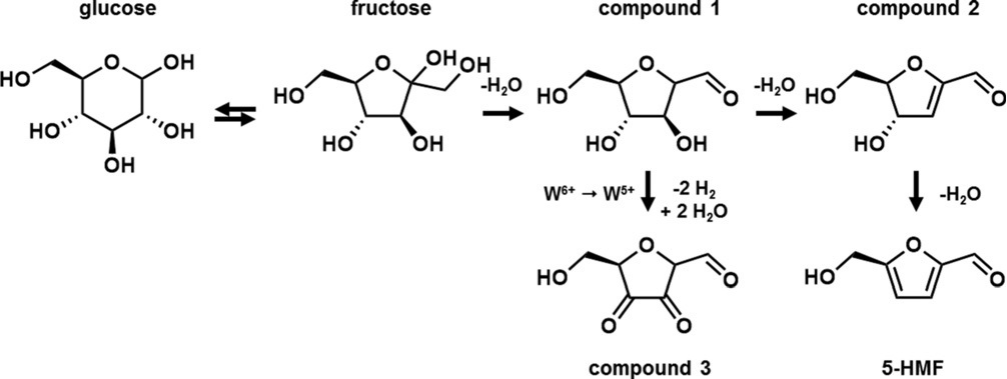
Reaction intermediates in the dehydration of glucose to HMF.

Finally, we attempted to identify the nature of the unknown reaction intermediate(s). For this purpose, we analyzed the reaction mixtures after 3 h using P/WO_3_ as the catalyst using LC‐ESI‐MS (see the Supporting Information). We observed the presence of HMF, two compounds with molecular masses of 144 g mol^−1^ and 158 g mol^−1^, unconverted sugars as monomers and oligomers (i.e., with other sugars, HMF, and the 144 g mol^−1^ compound), and 1,5‐anhydroglucitol. In the cyclic reaction mechanisms that are usually considered for fructose dehydration, the reaction of ketofuranose starts with dehydration of the hemiacetal at the C2 position, followed by two consecutive β‐dehydrations leading to HMF (Scheme 1). Both of these intermediates (compound 1 and compound 2) have earlier been identified by ^13^C NMR.^23^, ^24^ The ESI‐MS spectra suggest that the 144 g mol^−1^ compound is the product of the first two dehydration steps of fructose (compound 2 in Scheme 1). This would be (4*R*,5*R*)‐4‐hydroxy‐5‐hydroxymethyl‐4,5‐dihydrofuran‐2‐carbaldehyde, which has been identified as an intermediate in the dehydration of fructose to HMF in DMSO.^23^ The ESI‐MS spectrum of the 158 g mol^−1^ compound (compound 3 in Scheme 1) suggests that it is the dehydrogenation product of compound 1, i.e., one of the 2,5‐anydro‐hexoses that have also been implicated in fructose dehydration.^34^, ^35^, ^36^, ^37^ It is likely that this dehydrogenated product is a side‐product associated with the stoichiometric oxidation of the alcohol groups by the tungsten oxide catalyst into the stable dehydrogenated side‐product. In keeping with this, we observed the yellow WO_3_ sample turn blue, indicative of the reduction of W^6+^ to W^5+^. We speculate that this product is relatively stable, whereas compound 2 will be rapidly dehydrated to HMF by HCl.

## Conclusions

Mixed niobium–tungsten oxides were obtained by precipitation from aqueous solutions of NbCl_5_ and WCl_6_. The samples had the tungstite structure and at a high Nb content also contained niobic acid. The Brønsted and Lewis acidic properties of these oxides were compared to those of Nb_2_O_5_ by CO IR spectroscopy. Mixed Nb_*x*_–WO_3_ and WO_3_ catalyze the dehydration of glucose to HMF in water with good yield. The mixed Nb–W oxides have much lower Brønsted acidity than Nb_2_O_5_ and consequently, fewer by‐products were formed during glucose conversion. The optimum HMF yield was obtained for mixed oxides with balanced Lewis and Brønsted acidity. Although glucose is rapidly converted by tungstite in THF/water, the HMF product is formed slowly. Isomerization and dehydration occur at a high rate on the LAS and the weak BAS of WO_3_ and tungsten‐rich mixed oxides However, the last dehydration step to HMF requires stronger Brønsted acidity. An intermediate with a molecular mass of 144 g mol^−1^, likely (4*R*,5*R*)‐4‐hydroxy‐5‐hydroxymethyl‐4,5‐dihydrofuran‐2‐carbaldehyde, was observed by LC‐ESI‐MS. When HCl is added to the resulting reaction mixture, the HMF yield is about 56 % irrespective of the Nb content of the tungstite samples, confirming the slow dehydration of the partially dehydrated reaction intermediate. DFT calculations confirm that the tungstite surface can adsorb glucose, abstract a proton from glucose, and catalyze the hydride shift to fructose. Nb substitution in the tungstite surface structure stabilizes the deprotonated glucose intermediate and, accordingly, lowers the overall activation barrier for glucose isomerization. The present study suggests a novel strategy of combining Lewis acidic transition metal oxides with weak Brønsted acid sites to catalyze isomerization and dehydration to relatively stable intermediates followed by further dehydration by a mineral acid to obtain HMF.

## Supporting information

As a service to our authors and readers, this journal provides supporting information supplied by the authors. Such materials are peer reviewed and may be re‐organized for online delivery, but are not copy‐edited or typeset. Technical support issues arising from supporting information (other than missing files) should be addressed to the authors.

Supplementary
