# Supplementary material for: Dehydration of Glucose to 5‐Hydroxymethylfurfural Using Nb‐doped Tungstite
Source: ChemSusChem. 2016 Aug 5;9(17):2421–9. doi: 10.1002/cssc.201600649 (PMC5129542; doi:10.1002/cssc.201600649)

CHEMISTRY & SUSTAINABILITY
CHEMSUSCHEM
ENERGY & MATERIALS

Supporting Information

Dehydration of Glucose to 5-Hydroxymethylfurfural Using Nb-doped Tungstite

Chaochao Yue,[a] Guanna Li,[a] Evgeny A. Pidko,[a] Jan J. Wiesfeld,[a] Marcello Rigutto,[b] and Emiel J. M. Hensen*[a]

cssc_201600649_sm_miscellaneous_information.pdf

Table S1: Integrated areas of ESI-MS peaks of 5-HMF and the two compounds with m/z = 144 g/mol and m/z = 158 g/mol which are all products of the dehydration of glucose over WO_3 in a THF/water mixture at 120°C.

Reaction time (h)	m/z = 158	Hydrated HMF m/z = 144	HMF
0.5	125	18	n.d.
1	389	61	1012
2	407	144	1474
3	436	147	2596
3+HCl	319	48	6162

Figure S1: ESI-MS LC chromatograms of reaction mixtures obtained from glucose dehydration over WO_3 in a THF/water mixture at 120°C.

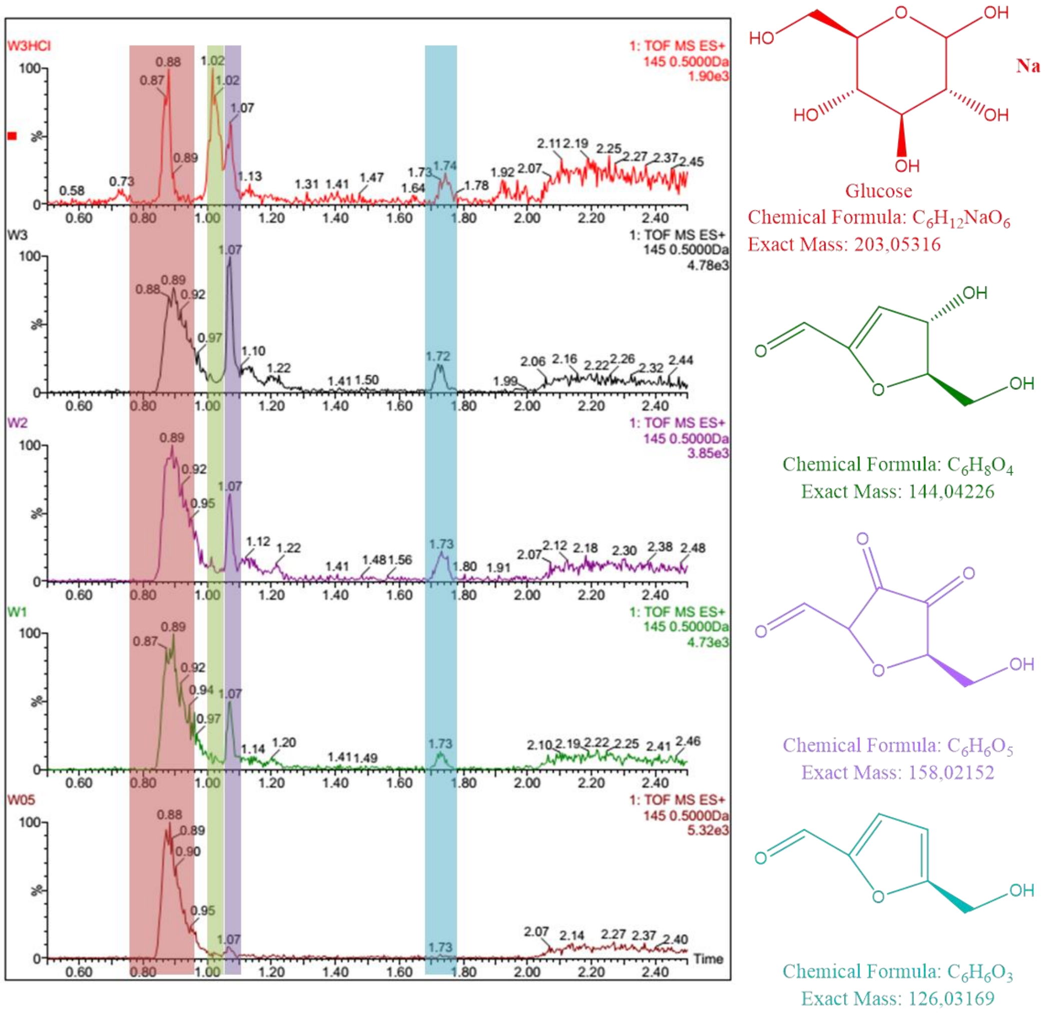

Supplement: Supplementary file 1 — Supplementary [file CSSC-9-2421-s001.pdf]
